# Preserving Microbial Community Integrity in Oilfield Produced Water

**DOI:** 10.3389/fmicb.2020.581387

**Published:** 2020-10-19

**Authors:** Natalie M. Rachel, Lisa M. Gieg

**Affiliations:** Petroleum Microbiology Research Group, Department of Biological Sciences, University of Calgary, Calgary, AB, Canada

**Keywords:** sample preservation, microbial community composition, oilfield microbiology, 16S rRNA gene, oilfield produced water

## Abstract

Determining a representative microbial signature from any given location is dependent on robust sample collection and handling. Different sampling locations and hence sample properties can vary widely; for example, soil would be collected and handled differently compared to liquid samples. In the event that sample material has a low concentration of biomass, large quantities need to be collected for microbial community analysis. This is certainly the case when investigating the microbiology of oilfield systems, wherein produced water (PW) is one of the most common sources for microbial sampling. As the detrimental effects of microbial metabolism within these industrial milieus are becoming increasingly well-established, the characterization of microbial community composition using molecular biological analyses is becoming more commonplace for accurate monitoring. As this field continues to develop, the importance for standardized operating protocols cannot be understated, so that industry can make the most informed operational decisions possible. Accurately identifying oilfield microbial communities is paramount, as improper preservation and storage following sample collection is known to lead to erroneous microbial identifications. Preserving oilfield PW can be challenging, as many locations are remote, requiring lengthy periods of time before samples can be processed and analyzed. While previous studies have characterized the effects of various preservatives on concentrated, filtered, or purified microbial samples, to the best of our knowledge, no such study has been undertaken on low biomass liquid samples. To this end, we investigated the effectiveness of nine different preservation conditions on PW collected from the same sampling location within a heavy-oil producing field, and monitored how the microbial community changed over the period of a month. Our results reveal that the choice of preservative drastically affects microbial community, and should be selected with careful consideration before sampling occurs.

## Introduction

Molecular techniques have become an invaluable biotechnological staple, with high-throughput sequencing (HTS) being particularly powerful at providing genetic insights into microbial communities (Hirsch et al., 2010; Caporaso et al., 2011; Mason et al., 2014). This now commonplace approach has expedited the identification and detection of unculturable microorganisms, making it feasible to comprehensively analyze complex environmental samples, such as those taken from petroleum reservoirs (Orphan et al., 2000; Grabowski et al., 2005; Duncan et al., 2009; Pham et al., 2009; Bonifay et al., 2017; Zhu and Al-Moniee, 2017). In oilfield systems, where the presence of certain types of microorganisms can contribute to infrastructure complications, such as corrosion, souring, and biofouling (Zuo et al., 2004; Coetser and Cloete, 2005; Enning et al., 2012; Basafa and Hawboldt, 2019), it is important to have a clear understanding of these potentially detrimental microorganisms. Produced waters (PW) are one of the most common sampling sources within oilfield systems, but are often characterized by low biomass and diversity (Oldham et al., 2019). To compensate, large volumes are collected, consequently introducing an additional experimental step requiring concentration of the biomass for downstream DNA extraction and microbial community member identification using molecular methods. Ideally, this processing should occur immediately after sample collection, but logistical constraints frequently prevent on-site DNA extraction. For example, many crude oil recovery operations are in remote locations and typically not equipped with processing equipment (such as filtration capabilities or mobile in-field DNA extraction and PCR units). As a result, PW is often transported to a laboratory for processing which can take several days (or longer) if the sampling location is particularly remote (Voordouw et al., 2016; De Paula et al., 2018). Under such conditions, substantial changes in microbial community composition are known to occur as samples are often exposed to different selective pressures (such as temperature and/or redox conditions; Kilbane, 2014; De Paula et al., 2018). This lag between sampling and processing time demonstrates the value in employing a preservation method to help maintain microbial community integrity. The effectiveness of a chemical nucleic acid preservation agent or protocol (such as refrigeration or freezing) has been previously evaluated on several different types of environmental samples, including filtered freshwater (Majaneva et al., 2018) and filtered seawater samples (Oldham et al., 2019), solid biological samples (Vink et al., 2005; Gray et al., 2013), soil (Lauber et al., 2010; Wallenius et al., 2010), sediments (Rissanen et al., 2010), and phytoplankton (Mäki et al., 2017). While general guidelines exist for obtaining microbiological samples from oilfield systems (Jack, 2002; NACE, 2014, 2018), specific protocols for sampling and preservation for HTS are lacking. This knowledge gap hinders the development and acceptance of a consensus for sample handling within the hydrocarbon energy industry, yielding discrepancies that could ultimately affect operational decisions. In addition, the lack of consensus approaches makes comparing results between studies difficult; indeed, a recent study that processed PW samples preserved in different ways and through two separate laboratory pipelines yielded different results (De Paula et al., 2018).

To help address these gaps, we conducted a comparative investigation to determine to what extent microbial community will change in the presence or absence of different preservatives or preservation strategies over time. For the study, we specifically tested the sampling case wherein on-site filtering or other PW sample processing would not be possible and wherein samples could be stored for up to month prior to HTS analysis in a laboratory. Seven different chemical preservatives that were added directly to unfiltered PW were assessed in the study. These included alcohols [95% ethanol (ETOH) and 70% isopropanol (IPA), Everclear®, a homemade preservation solution DMSO-EDTA-salt (DESS); Gray et al., 2013], and three commercially-available proprietary nucleic acid preservatives (DNAzol®, DNAgard®, and RNAlater™). In addition, two different storage temperatures (−20 and 4°C) were employed (in the absence of chemical preservative). Preserved samples were compared to unpreserved aliquots handled in the same manner as their preserved counterparts. Our results indicate that while some deviation from the initial community composition was observed (particularly in the relative abundances of some taxa), and the alcohols, DESS, and temperature control tests were the most effective at maintaining microbial community integrity of the sampled PW under the tested conditions. These findings will help contribute to establishing the much-needed standardized operating protocols for future handling of PW and other liquid samples meant for downstream molecular microbiological analysis.

## Materials and Methods

### Sample Collection and Processing

The oilfield selected for PW sampling was the Medicine Hat Glauconitic C field, a low temperature (26–30°C), low salinity, and heavy-oil field undergoing nitrate treatment for souring control, located near Medicine Hat, Alberta, Canada (Voordouw et al., 2009; Shen et al., 2018). This field was selected based on its close proximity to the laboratory so that collected samples could be initially processed within a few hours of collection. One production well was sampled for the experiment (18-PW), selected as it is known to harbor an active microbial community (Fida et al., 2016; Suri et al., 2019) and to ensure that the compared samples originated from the same source at the same point in time. For each of the nine preservation conditions, two sterilized bottles were prepared: one wide mouth, 1-L polypropylene (VWR International), and one accompanying 500 ml Nalgene® media bottle (Style 2020, Sigma Aldrich), totaling 18 bottles for the entirety of sampling. In preparation for collection, prior to departure, an appropriate volume of each chemical preservative was added to one of the 500-ml bottles as indicated in Table 1. The amounts of commercially-available preservatives added were based on the volumes recommended by the manufacturers. In the absence of data or guidelines, a 20% v/v of the alcohols or DESS was chosen as the experimental value to achieve preservation without over-diluting the sample such that insufficient nucleic acid yields would occur. If samples contained sufficient biomass to allow for a greater volume to be added, then it was possible that any preservative properties could have been enhanced; however, while this hypothesis is of interest, it goes beyond the scope of this study and was not explored here. The DESS solution was prepared by combining 0.25 M disodium EDTA (pH 8.0), 20% dimethyl sulfoxide in a saturated NaCl solution (90 g in 500 ml) as described by Gray et al. (2013). Each 1-L bottle was used to capture PW directly from the wellhead following initial purging. After the 1-L bottle had been filled, its preservative-containing, accompanying 500 ml bottle was then immediately filled from that same bottle to a final volume of 600 ml (just below the cap). This was to ensure that the PW used for both the unpreserved control (UPC), which is the leftover ~400 ml in the 1-L bottle, and PW used in the preserved sample were identical, reducing the possibility of variations between control and the experimental samples as much as possible. ATP, as a measure of microbial activity, was quantified on-site using a luminometer and a commercially-available kit and protocol (OSP Microcheck, Calgary, Canada). This approach gave a cell number of 1.5 × 10^5^ (+/− 2.4 × 10^4^) cells/ml PW or 1.5 × 10^8^ cells/L of the sampled PW.

**Table 1:** Details and concentrations of chemical preservatives used to treat produced water (PW) samples in this study.

Preservative	Supplier	Volume added (ml)	Final concentration
95% ETOH	University supply	126	20% (v/v)
Everclear® (95%)	Luxco, Inc.	126	20% (v/v)
70% isopropanol	Equate™	171	20% (v/v)
DNAzol®	Invitrogen	3.6	1 ml per 2.5 × 10^7^ cells (Recommended: 1 ml per 1–3 × 10^7^ cells)
RNAlater™	Invitrogen	1.8	1 ml per 5 × 10^7^ cells (Recommended: 0.5–1 ml per 3 × 10^8^ cells)
DNAgard™	Biomatrica	3.6	1 ml per 2.5 × 10^7^ cells (Recommended: 1 ml per 1 × 10^7^ cells)
DESS	General chemical supplier (Fisher Scientific)	120	20% (v/v)

All samples were transported to the laboratory within 4 h of collection at ambient temperature (~23°C), with the exception of samples that were later refrigerated or frozen; these were kept on ice during transport. All Day 0 samples (*n* = 34) were then immediately processed for microbial community analysis. These 34 samples included triplicates of the nine preservation conditions examined, as well as one respective UPC for each condition. The 4°C and freezing samples were exceptions and went without a separate Day 0 control. As no additive was present in these samples and they were only subjected to temperature control upon arrival at the laboratory, an UPC was considered redundant. Samples were then stored for up to 28 days, with sub-samples collected after 7, 14, and 28 days for DNA extraction and amplicon sequencing. All samples containing the chemical preservatives were stored at room temperature, with the exception of the two experimental samples for which the preservation conditions entailed storage at 4 and −20°C.

### DNA Extraction

Samples were filtered using sterile 0.2 μm polyethersulfone (PES) filter units (Thermo Scientific) to concentrate biomass; the volume filtered for each sample and time point varied depending on how quickly the filter became clogged with biomass (Supplementary Table S1). The PES filter was excised using sterile stainless steel surgical blades, cut into small pieces within a sterile Petri dish, and transferred to a sterile microfuge tube containing lysis matrix for homogenization. Cut filters containing preserved PW biomass were evenly distributed among three lysis tubes, and all subsequent molecular manipulations were carried out individually for each tube. This was to prepare triplicates of the DNA extractions. Genomic DNA extraction was carried out using the FastDNA™ SPIN Kit for Soil (MP Biomedicals), with an additional digestion step: after homogenization, 10 μl of Proteinase K (20 mg/ml) and 10 μl of 10% SDS were added to the lysate. After vortexing, the mixture was incubated for 30 min at 50°C. Extraction then continued using the standard protocol provided by the manufacturer. DNA concentrations were quantified using a Qubit® fluorometer (Invitrogen).

### Microbial Community Analysis

The V4-V5 region of the 16S rRNA gene was amplified using the primers 515F (5' GTGYCAGCMGCCGCGGTAA) and 926R (5' CCGYCWATTYMTTTRAGTTT; Walters et al., 2015; Parada et al., 2016). The amplification protocol was as follows. Using KAPA HiFi HotStart ReadyMix PCR Kit (KAPA Biosystems), 25 μl reactions were prepared containing 0.3 μM of each primer and 10 ng of genomic DNA, as suggested by the manufacturer. For samples that yielded lower quantities of genomic DNA, a volume of up to 11 μl of extracted DNA was added to the PCR reaction. Amplicons were purified using AMPure XP magnetic beads (Beckman Coulter), quantified, and subsequently used as template for a second round of PCR to attach index primers. Indices were from a Nextera XT v2 Kit (P5-S50X-OHAF and P7-N7XX-OHAF, Illumina) and added to a reaction mixture containing Taq polymerase PCR Master Mix (Thermo Scientific), 0.3 μM primer, and 5 ng of purified amplicon DNA to a final volume of 50 μl. Thermocycling conditions are shown in Supplementary Table S2. Amplicons containing indices were purified using a QIAquick® gel extraction kit (Qiagen) and quantified. Subsequent steps were carried out at the Centre for Health Genomics and Informatics (University of Calgary, AB, Canada). Library purity was assessed using a 2200 TapeStation system (Agilent). HTS was done using a paired-end, v2 300 cycle sequencing kit for Illumina MiSeq.

Primers were removed from raw reads using CutAdapt (Martin, 2011), and sequencing trimming and read quality was assessed using DADA2 (Callahan et al., 2016). Sequencing data analysis and reads with a quality threshold of *q* = 30 were assembled using the default parameters of QIIME2 version 2019.7 (Bolyen et al., 2019). Taxonomy was assigned by classifying against the SSU SILVA database version 132 (Quast et al., 2013; Yilmaz et al., 2014), and analyses were performed based on the lowest taxonomic level identified. The QIIME-assigned abundances were analyzed using non-metric multidimensional scaling (NMDS) in R with the Vegan package (Version 2.5-6). Plots were generated in ggplot2 under default parameters (Wickham, 2016) using RStudio version 1.2.5001 (RStudio Team, 2019). Unweighted UniFrac distances (Lozupone and Knight, 2005) and beta diversity statistics were analyzed using PERMANOVA (Anderson, 2001) using the beta-group-significance command in QIIME2. Gene sequences were deposited in GenBank, under the BioSample accession number SAMN15480607.

## Results

### Establishing a Taxonomic Baseline

In order to compare the effects of storage on microbial community composition, we began by analyzing the characteristics of the PW samples (*n* = 34) processed the same day they were collected (Day 0). The microbial community compositions of all other preserved samples (e.g., after 7, 14, or 28 days of storage) were compared to those of Day 0, as well as to their respective UPC sample stored for the same period of time.

**Figure 1:**
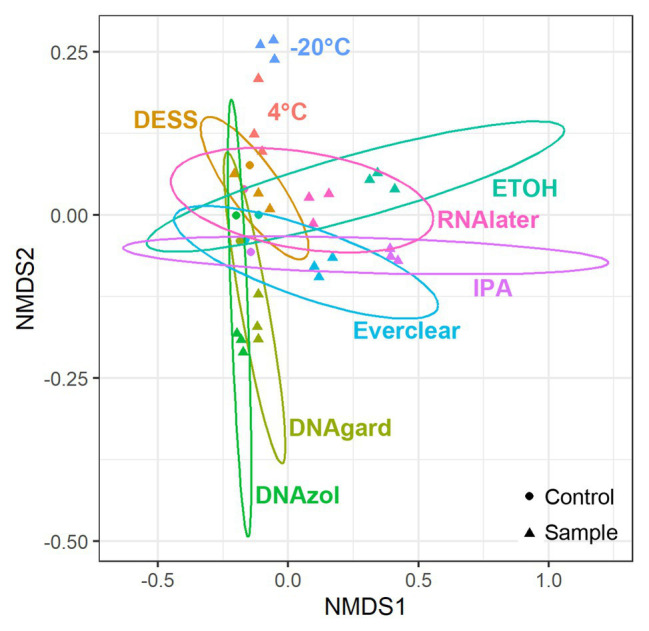
Non-metric multidimensional scaling (NMDS) plots showing taxonomic similarities for all samples at Day 0. Stress value = 0.083.

As we were interested in evaluating community similarity between samples, an ordination technique designed to analyze and visualize patterns within large, multivariate data sets was used. Specifically, a NMDS analysis was applied to the sequencing data, as this technique uses non-Euclidean dissimilarity or distance matrices; in this study, Bray-Curtis dissimilarity was utilized (Bray and Curtis, 1957) to evaluate microbial community composition differences among separate populations/samples. Ultimately, the hundreds of different taxonomic variables present in each PW sample were distilled such that those having similar microbial community composition will co-locate, making it possible to detect patterns of dissimilarity. NMDS plots were structured according to differences in preservative and sample type (UPCs or PW containing preservative). When compared, the microbial community composition of all Day 0 samples yielded several observations (Figure 1): first, triplicates of the preserved samples, regardless of the preservative used, clustered closely together, indicating that the triplicates were reproducible. At the same time, the UPCs also clustered together, but away from their respective preserved samples. This indicates that even within the hours of collection and processing, there were notable differences between the UPCs and preserved samples. These differences could be due to the fact that the microbial community in the unpreserved samples changed slightly between the time of sample collection and transport to the laboratory for processing. The one exception to this observation was for the PW sample containing DESS, which clustered closely with its UPC. A possible explanation for this result could be that the preservation did not take effect immediately, causing the community within the DESS-treated samples to continue to change in a similar manner as its UPC.

**Figure 2:**
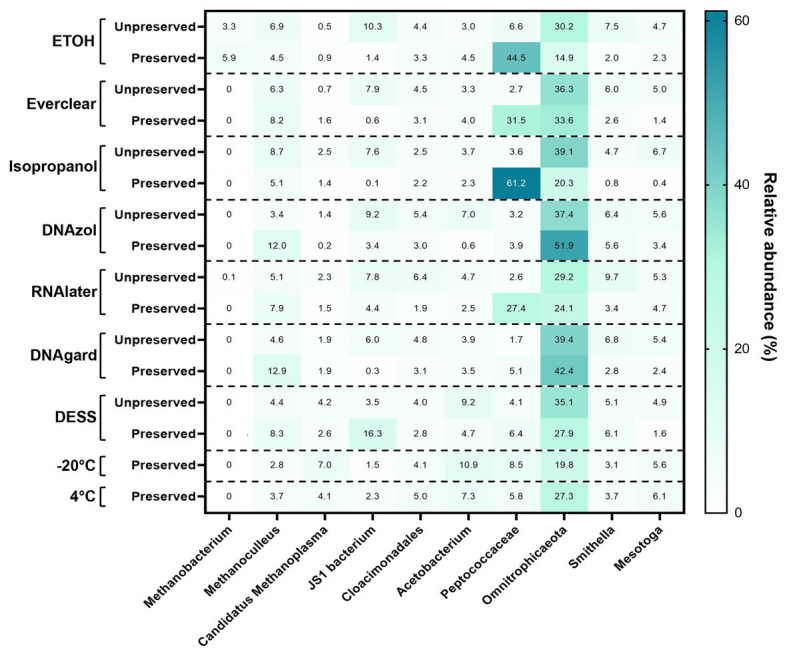
Heat map summarizing the relative abundance of the top 10 taxa detected within all Day 0 samples.

To pinpoint the source(s) of these differences and to obtain an encompassing picture of the microbial communities at Day 0, a detailed taxonomic breakdown was performed. The relative abundances of the top 10 most abundant microbes were plotted (Figure 2; Supplementary Table S3). Notably, *Omnitrophicaeota* was abundant in all samples. *Peptococcaceae* was also predominant in several of the preserved samples, including those preserved with 95% ethanol, Everclear, 70% isopropanol, and RNAlater™. Other samples had less dramatic, but visible microbial signatures, varying in abundances. For example, the methanogen *Methanoculleus* was consistently observed, and was present in PW treated with DNAgard or DNAzol by a three- or four-fold excess compared to their UPCs. A sequence closely affiliating with JS1 bacterium was four-fold in excess for PW preserved with DESS. These relative abundance differences likely account for the differences observed in the NMDS plot. Overall, the taxonomic breakdown (Figure 2) is consistent with the patterns seen in the NMDS plot (Figure 1).

It is worth noting that samples stored at −20 and 4°C did not have a separate UPC at Day 0; since temperature control takes time to achieve and does not entail the immediate effect of the addition of a chemical, a Day 0 UPC would be redundant. These samples were kept chilled on ice during transport, until arriving in the laboratory for proper storage and processing.

Once a taxonomic baseline was established, comparisons were then made to evaluate the effectiveness of the various preservation approaches following different lengths of storage. This was done by analyzing the microbial community compositions at various time points with respect to the Day 0 baseline, and to their respective UPC. If effective, we would expect a similar microbial community composition between Day 0 UPC and the preserved sample, indicating that there has not been a substantial shift over time. The same processing and analysis carried out for the Day 0 samples was performed after 7, 14, and 28 days of storage of the same samples.

### Alcohols and DESS

**Figure 3:**
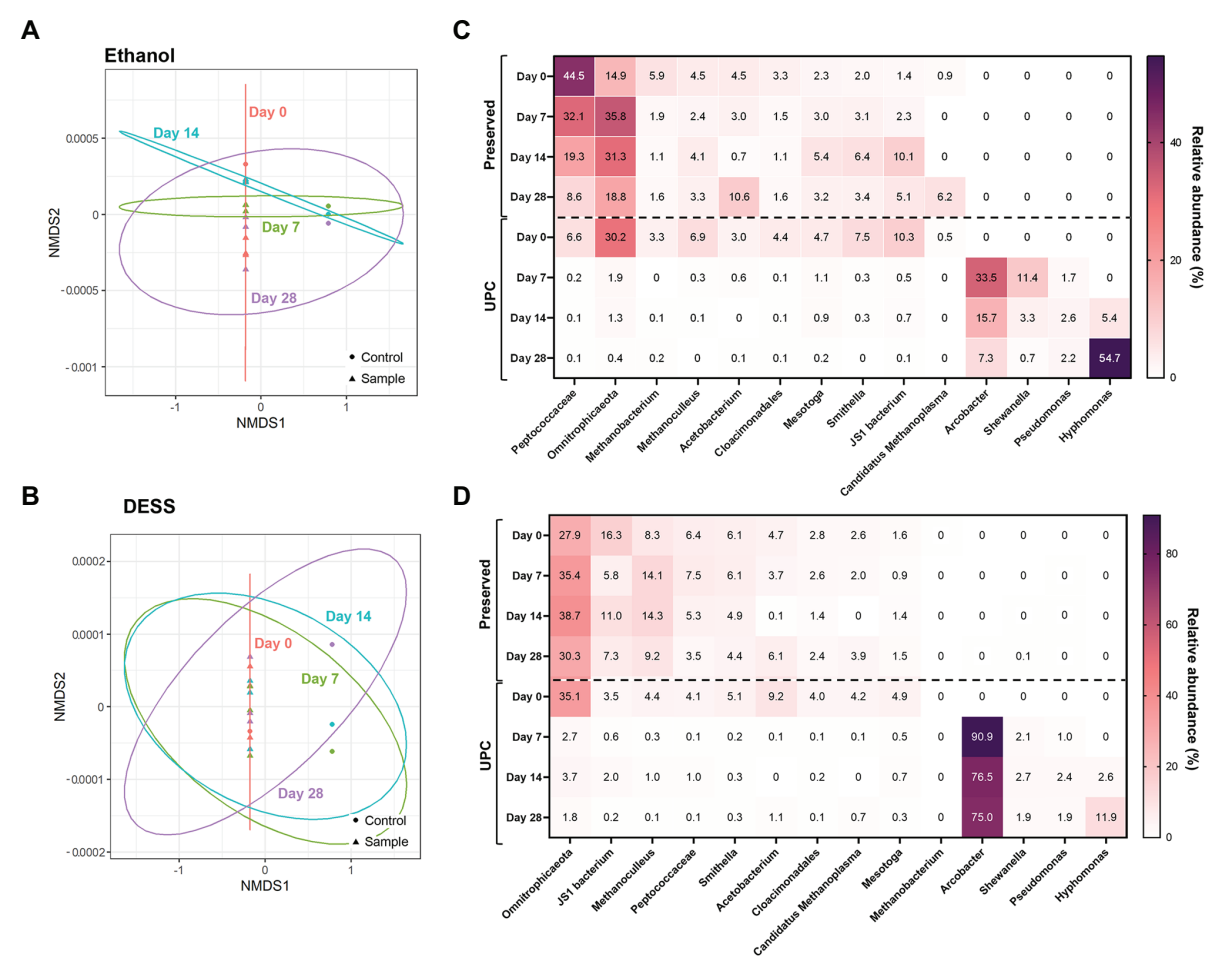
NMDS **(A,B)** and taxonomic heat maps **(C,D)** of representative samples treated with non-proprietary preservatives: ethanol (**A**,**C**; stress value = 1.4 × 10^−4^) and DESS (**B**,**D**; stress value = 9.6 × 10^−5^).

After subjecting the data to NMDS, a pattern among PW treated with non-proprietary chemical preservatives was observed (Figure 3; Supplementary Figure S1). All experimental triplicates treated with 95% ethanol, Everclear®, 70% isopropanol, or DESS clustered closely with their Day 0 UPC. In contrast, the corresponding UPCs following storage after Day 7, Day 14, and Day 28 deviated substantially from the Day 0 samples as seen on the NMDS plots (Figure 3; Supplementary Figure S1). This result shows that the PW samples preserved with alcohols or DESS and then stored were similar in community structure to their Day 0 microbial composition. In addition, differences between community composition of alcohol-treated samples and those treated using other preservatives were found to be significant, with combinations of isopropanol with Everclear and freezing storage being exceptions (Supplementary Table S4). Numerous DESS-treated samples were also found to not have statistically significant differences with the communities present in Everclear, isopropanol, freezing, or cold storage. This result supports the patterns observed with NMDS plots, indicating similar community compositions for these samples. Preserved samples with similar NMDS patterns but calculated to have significant community differences were also observed, such as between ethanol and Everclear (Figure 3; Supplementary Figure S1). Therefore specific taxonomic composition can vary, but still retain an overall common community structure. Taxonomic analysis revealed some changes in relative abundances of specific taxa compared to the Day 0 microbial community compositions, and these changes were different depending on the preservative. For example, in the 95% ethanol-preserved samples, the relative abundances of *Peptococcaceae* decreased with increased storage time (change from 45 to 8.6% relative abundance; Figure 3C), while a lesser effect was seen with this taxon when DESS was used as the chemical preservative (Figure 3D). In addition, the addition of an alcohol preservative altered the relative sequence abundance of some taxa at Day 0 compared to its UPC at Day 0. For example, all UPCs at Day 0 revealed *Peptococcaceae* to be present in comparatively low relative abundances (2.7–6.6%); however, the addition of 20% vol/vol alcohol increased this relative abundance substantially (to 31.5–61.2%; Figure 3C; Supplementary Figures S1C,D). In contrast, this effect was not observed with DESS as the chemical preservative (Figure 3D). Despite these differences in relative abundance values, the microbial community compositions in the UPC samples stored for 7, 14, and 28 days underwent a more dramatic shift, with four taxa not previously detected (<0.1% relative abundance) becoming predominantly abundant, including sequences affiliating with *Arcobacter*, *Pseudomonas*, *Shewanella*, and *Hyphomonas* (Figure 3; Supplementary Figure S1). Taken together, these results showed that the presence of 95% ethanol, Everclear®, 70% isopropanol, and DESS reasonably aided the preservation of the original PW microbial community for up to 28 days of storage.

### Temperature Control

Produced water stored by either refrigeration or freezing displayed similar patterns to those treated with the alcohols or DESS (Supplementary Figure S2). In the absence of preservation under cold temperatures, *Arcobacter* overwhelmed the microbial community structure, with *Hyphomonas* increasing in relative sequence abundance after 28 days (Supplementary Figure S2C). Instead of forming two distinct clusters, three were observed. When stored at 4°C, unlike what was observed for non-proprietary treated solutions, the Day 0 samples clustered separately (Supplementary Figure S2A). Lower relative abundances for *Omnitrophicaeota* and *Peptococcaceae* at Day 0 could be the reason for these dissimilarities, as no stark changes were observed based on the detailed taxonomic breakdown. UPCs and 4°C-preserved samples remained in their own, tightly-concentrated clusters. Similar for samples stored at −20°C, UPCs and preserved samples clustered together, including Day 0, reflecting the differences between these two population sets (Supplementary Figure S3). Samples stored at −20°C for 28 days showed an overabundance of *Shewanella*, which was not observed at the other time points, nor in the UPCs. These results suggest that temperature control also aids in community preservation, with −20°C being preferable, and 4°C is less effective than the presence of one of the four non-proprietary solutions.

### Commercially-Available Proprietary Preservatives

**Figure 4:**
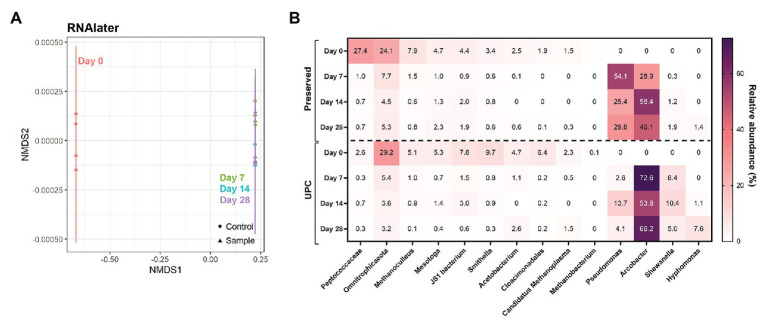
NMDS **(A)** and taxonomic heat map **(B)** of representative samples treated with chemical preservatives: RNAlater. Stress value = 7.7 × 10^−5^.

Samples treated with commercially-available nucleic acid preservatives presented dramatic differences within their microbial community compositions with prolonged storage. RNAlater™-treated samples, for example, showed a high community similarity between its UPC and Day 0 triplicates (Figure 4). However, after storage for 7 days or longer, all samples clustered tightly with their corresponding UPCs, indicating that the treated samples were subject to the same transformation as their untreated controls (Figure 4A). Upon examination of the taxonomic breakdown, *Pseudomonas* and/or *Arcobacter*, not detected at >0.1% relative abundance in the Day 0 samples, dominated the microbial community profiles after 7 days of storage in both population sets (Figure 4B). Samples treated with DNAzol® or DNAgard® also showed a similar divergence from their Day 0 taxonomic baseline, albeit they formed three distinct clusters (Supplementary Figure S4). UPCs and Day 7, 14, and 28 samples co-located to their own clusters; despite that both had enrichments of specific taxa in their community profiles, taxon identities varied. Both UPCs for DNAzol® and DNAgard® became enriched in *Arcobacter* and later *Hyphomonas*, whereas the treated samples contained *Shewanella* and *Pseudomonas*. These results illustrate that under the testing conditions, the commercially-available chemical preservatives were not suitable for preserving the unfiltered PW sample for long term storage at room temperature.

## Discussion

Proper handling technique of biological samples is essential for reproducible and reliable microbial community identifications, especially with environmental samples that can be difficult to obtain and sensitive to changing conditions (such as temperature and redox status). While experimental processing, such as DNA extraction and downstream molecular steps, is often rigorous, the same degree of rigor can be easily overlooked during sample collection. Unlike with molecular techniques, where nucleic acid quantity and purity can be readily assessed, it is difficult to determine whether a field sample has been effectively preserved. Furthermore, in the absence of knowledge pertaining as to what occurs and to what extent with treated samples, assumptions are potentially made that one handling technique will transfer effectively to samples with entirely different properties. Here, we sought to shed light on this knowledge gap, so that effective sample handling protocols may be amended and established specifically for oilfield PW or other aqueous, low-biomass (≤10^4^ cells/ml) environmental samples. While our study is not exhaustive, our goal was to focus on reproducibility and variety, both in terms of preservation conditions and time points, so that it may be favored to produce convincing results.

The decision to incubate samples as-is, unfiltered, at room temperature, was made to address sample handling from a “worst case scenario” perspective. Additional steps such as filtering and storage at a cool temperature contribute to preserving the integrity of the microbial community; indeed, previous work has addressed such considerations (Jack, 2002; Majaneva et al., 2018; Oldham et al., 2019). However, we were interested in investigating the extent to which microbial community composition would be affected without additional steps such as filtering, and in the event that prolonged refrigerated storage was not an option. In addition, two of the chemical preservatives, Everclear® and general purpose 70% isopropanol, were selected because they are not intended for research purposes, but are easily accessible and in the case of isopropanol, inexpensive. In the end, these “off-the-shelf” alcohol solutions were as effective as laboratory-grade 95% ethanol in preserving microbial community composition under storage conditions.

The prevalence of *Peptococcaceae* in some preserved samples at Day 0, but not all, nor their UPCs, is unclear. We attempt to explain this finding by considering that in the presence of salt, the solubility of DNA in alcohol is poor; with sufficient concentrations of alcohol and salt, DNA will precipitate. When due for processing, the PW samples were all filtered owing to their large volume; precipitated DNA will be more likely to be retained within the biomass that accumulates on the filter than solubilized DNA. As *Peptococcaceae* are obligate anaerobes (Stackebrandt, 2014), they may have lysed shortly after sample collection or during transportation, releasing their DNA into solution. Their relative abundances do decrease as incubation time increases (Figure 3), but this could reflect the gradual change of other microorganisms that remain intact within the PW. Arguably, a final concentration of 20% alcohol (v/v) may not have been sufficient to cause precipitation, but as the samples are PW containing other substances, such as hydrocarbons, minerals, and salts, these other factors may have aided preservation and/or contributed to preservation results. This is supported by the fact that this result is also observed with RNAlater™, which drastically increased the ammonium concentration of the PW (Supplementary Table S5). The additional ammonium may also have been sufficient to precipitate the DNA, if the dielectric constant of the PW was sufficiently low enough for an interaction to occur. These hypotheses remain unproven, but even so, it highlights the potential peril of losing DNA when filtering liquid samples for DNA extraction. In addition, there is evidence demonstrating that preservation agents are more impactful towards the relative abundance of taxa, rather than their presence or absence (Menke et al., 2017; Oldham et al., 2019). This data support our findings as well, and may be an unavoidable consequence of preservation, highlighting the value of replicates and consistency for treatment.

The overabundance of *Shewanella* in the Day 28 samples that had been stored at −20°C was also unclear (Supplementary Figure S3); the source of this discrepancy is difficult to explain. Cross-contamination seems unlikely, as the triplicates were reproducible, and no other sample revealed such a high relative abundance of *Shewanella* their microbial community profiles. *Shewanella* is incredibly robust with respect to temperature, and can be propagated at temperatures close to 0°C, displaying numerous unusual physiological changes to compensate (Abboud et al., 2005). Prior to processing, the Day 28 samples had been frozen for 2 weeks, and thawed for a third time, having been done so twice for processing Day 7 and Day 14 time points. These freeze-thaw cycles along with the lengthy final frozen incubation period could have been disruptive to other microorganisms in the community that are not as resilient to cold, decreasing their relative abundance in comparison to *Shewanella*. In light of this finding, it may be beneficial to mix and evenly divide the sample into the appropriate number of aliquots for each time point to be assayed separately to prevent freeze-thaw cycles that can impact microbial community compositions.

The results of this study may have been influenced by both the characteristics of the PW sample and the concentrations/amounts of the chemical preservatives that were used. The PW chosen for the study was retrieved from a low temperature reservoir (downhole temperature ~26–30°C) that is considered to be of low salinity; it is possible that samples from different reservoirs and/or having different characteristics (such as higher salinity) may be affected differently by the different chemical preservatives. We acknowledge that the final concentration of any given chemical preservative used is a variable that can impact its effectiveness, and may be a reason why the commercially-available, proprietary chemical preservatives proved to be ineffective with the unfiltered PW sample used here. While the volumes added to PW (Table 1) were based on cell counts as suggested by the manufacturer, their protocols for intended use do not involve dilution. Rather, the sample, such as tissue or a cell pellet, is to be (re)suspended directly into the solution. Other studies wherein DNAzol® was added to filters containing biomass (e.g., filtered water samples), showed it to be very effective in preserving microbial community integrity (Duncan et al., 2017; Oldham et al., 2019). Thus, proprietary nucleic acid preservatives such as the ones tested in the present study can be effective when used in a different manner. With respect to alcohols and DESS, a higher concentration may also be more effective at ensuring microbial community integrity; however, this increases the dilution factor of the liquid sample, which already contains a relatively low amount of biomass. Therefore, discretion by the researchers must be taken, as to what would be an appropriate concentration of preservative is necessary to achieve the desired effect, and may require some investigation prior to embarking on sample collection. In addition, alcohols may not be a feasible option depending on the sampling location, as they can be prohibited substances in some oilfield operations (e.g., offshore oil platforms). In this event, results suggest that DESS would be the best choice, although this requires laboratory preparation prior to sampling.

Overall, our results indicate that alcohols, DESS, and temperature control aided in preserving microbial community integrity within unfiltered PW when stored for up to 28 days post-sampling. Although similar taxa were observed at Day 0 and Day 28 when these preservatives were used, relative abundances changed for some taxa, thus the preservatives did have some limitations when added directly to unfiltered oilfield PW samples. Ultimately, we recommend that without previous research demonstrating preservative effectiveness for the type of samples that will be collected, a study should be established to investigate to help determine ideal conditions and concentrations. Reproducibility and comparability are critical pillars in scientific works, and ensuring proper sample treatment from the start is not a trivial consideration. The results communicated in this study will contribute to continued development of standardized protocols for accurately assessing microbial communities in oilfield environments.

## Data Availability Statement

The datasets presented in this study can be found in online repositories. The names of the repository/repositories and accession number(s) can be found in the article/Supplementary Material.

## Author Contributions

NR and LG conceived this study. NR performed all experimental procedures and bioinformatics analysis. The manuscript was written and edited by NR and LG. All authors contributed to the article and approved the submitted version.

### Conflict of Interest

The authors declare that the research was conducted in the absence of any commercial or financial relationships that could be construed as a potential conflict of interest.
