# Supplementary material for: Preserving Microbial Community Integrity in Oilfield Produced Water
Source: Front Microbiol. 2020 Oct 19;11:581387. doi: 10.3389/fmicb.2020.581387 (PMC7604316; doi:10.3389/fmicb.2020.581387)

SUPPLEMENTARY MATERIALS

Preserving Microbial Community Integrity in Produced Water

Natalie M. Rachel[1] and Lisa M. Gieg[1]

[1]Petroleum Microbiology Research Group, Department of Biological Sciences, University of Calgary, Calgary, AB, Canada

4 Supplementary Tables
4 Supplementary Figures

Table S-1. Volumes of PW filtered for DNA extraction.

Preservative	UPC	[DNA] ng/µL/mL	Sample	[DNA] ng/µL/mL
		**Day 0**		
**Preservative**	**UPC**	**[DNA] ng/µL/mL**	**Sample**	**[DNA] ng/µL/mL**
ETOH	~ 50 mL	0.042	~ 75 mL	0.009 ± 0
Everclear	~ 60 mL	0.02	~ 110 mL	0.004 ± 0.0006
Isopropanol	~ 40 mL	0.0125	~ 100 mL	0.004 ± 0.001
DNAzol®	~ 45 mL	0.024	~ 115 mL	0.027 ± 0.002
RNAlater™	~ 45 mL	0.013	~ 85 mL	0.008 ± 0.003
DNAgard™	~ 50 mL	0.01	~ 100 mL	0.005 ± 0.001
DESS	~ 50 mL	0.004	~ 120 mL	0.003 ± 0.001
-20°C	N/A	N/A	~ 70 mL	0.001 ± 0
4°C	N/A	N/A	~ 65 mL	0.003 ± 0.001
		**Day 7**		
**Preservative**	**UPC**	**[DNA] ng/µL/mL**	**Sample**	**[DNA] ng/µL/mL**
ETOH	~ 75 mL	0.029	~ 95 mL	0.008 ± 0.001
Everclear	~ 80 mL	0.01	~ 160 mL	0.002 ± 0.0003
Isopropanol	~ 100 mL	0.014	~ 110 mL	0.003 ± 0.003
DNAzol®	~ 95 mL	0.008	~ 125 mL	≥ 0.24 ± 0
RNAlater™	~ 75 mL	0.007	~ 510 mL	0.009 ± 0.001
DNAgard™	~ 90 mL	0.004	~ 130 mL	0.19 ± 0.012
DESS	~ 100 mL	0.006	~ 145 mL	0.003 ± 0
-20°C	~ 55 mL	0.007	~ 145 mL	0.007 ± 0.001
4°C	~ 50 mL	0.008	~ 170 mL	0.007 ± 0
		**Day 14**		
**Preservative**	**UPC**	**[DNA] ng/µL/mL**	**Sample**	**[DNA] ng/µL/mL**
ETOH	~ 50 mL	0.092	~ 100 mL	0.012 ± 0.06
Everclear	~ 65 mL	0.015	~ 120 mL	0.007 ± 0.001
Isopropanol	~ 100 mL	0.008	~ 100 mL	0.009 ± 0.001
DNAzol®	~ 95 mL	0.018	~ 160 mL	0.14 ± 0.050
RNAlater™	~ 100 mL	0.015	~ 275 mL	0.014 ± 0.001
DNAgard™	~ 100 mL	0.016	~ 130 mL	0.092 ± 0.026
DESS	~ 100 mL	0.027	~ 155 mL	0.007 ± 0.001
-20°C	~ 110 mL	0.015	~ 145 mL	0.010 ± 0.003
4°C	~ 110 mL	0.017	~ 145 mL	0.016 ± 0.002
		**Day 28**		
**Preservative**	**UPC**	**[DNA] ng/µL/mL**	**Sample**	**[DNA] ng/µL/mL**
ETOH	~ 190 mL	0.084	~ 100 mL	0.011 ± 0.05
Everclear	~ 190 mL	0.02	~ 170 mL	0.008 ± 0.001
Isopropanol	~ 165 mL	0.009	~ 145 mL	0.011 ± 0.001
DNAzol®	~ 100 mL	0.02	~ 140 mL	0.021 ± 0.032
RNAlater™	~ 180 mL	0.011	~ 335 mL	0.017 ± 0.002
DNAgard™	~ 140 mL	0.009	~ 155 mL	0.10 ± 0.038
DESS	~ 125 mL	0.022	~ 150 mL	0.008 ± 0.001
-20°C	~ 200 mL	0.019	~ 185 mL	0.012 ± 0.002
4°C	~ 195 mL	0.018	~ 205 mL	0.013 ± 0.001

Sample cell count
Measured ~1.5×10^5 cells/mL ± 2.4×10^4 based on ATP
= 1.5×10^8 cells/L of PW

DESS solution
0.25 M disodium EDTA pH 8.0
20% DMSO (Dimethyl sulfoxide)
NaCl saturated (~90g in 500 mL)

Table S-2. Thermocycling conditions.

KAPA Polymerase for 16S rRNA amplicon synthesis

Temperature (°C)	Time (s)	Cycles
95	180	1
98	20	
65	15	34
72	15	
72	60	1
4	∞	1

Taq for indices integration into 16S rRNA amplicons

Temperature (°C)	Time (s)	Cycles
95	180	1
95	30	
55	30	34
72	45	
72	300	1
4	∞	1

Table S-3. Error values of taxonomic relative abundances.

Day 0

	Ethanol		Everclear		Isopropanol		DNAzol		RNAlater		DNAgard		DESS		4C		Freezing	
Taxa ID	Average	σ	Average	σ	Average	σ	Average	σ	Average	σ	Average	σ	Average	σ	Average	σ	Average	σ
Peptococcaceae	44.5	4.18	31.5	3.5	61.1	1.7	3.8	0.7	27.3	3.0	5.0	0.3	6.4	2.1	8.5	1.9	5.8	0.8
Omnitrophicaeota	14.9	1.9	33.5	3.1	20.2	0.2	51.8	1.9	24.0	2.1	42.	2.3	27.9	3.4	19.8	0.5	27.3	2.7
Methanobacterium	5.8	0.9	0	0	0	0	0	0	0	0	0	0	0	0	0	0	0	0
Acetobacterium	4.4	0.8	3.9	1.9	2.2	0.4	0.6	0.3	2.5	0.3	3.4	0.7	4.7	0.9	10.9	0.4	7.3	2.3
Methanoculleus	4.4	0.4	8.2	3.1	5.1	0.5	11.9	1.1	7.8	1.0	12.8	2.0	8.3	3.0	2.7	0.6	3.6	1.3
Cloacimonadales	3.3	0.3	3.0	0.4	2.1	0.2	2.9	0.7	1.8	0.4	3.0	0.4	2.7	0.1	4.0	0.4	4.9	0.4
Mesotoga	2.3	0.3	1.4	1.1	0.4	0.3	3.4	0.7	4.6	0.7	2.4	0.2	1.5	1.1	5.5	1.0	6.1	0.3
Smithella	1.9	0.8	2.6	0.2	0.8	0.3	5.6	0.7	3.4	0.2	2.8	0.5	6.0	0.4	3.0	0.3	3.6	0.5
JS1 bacterium	1.3	0.6	0.6	0.1	0.0	0.0	3.3	1.3	4.4	0.4	0.2	0.1	16.3	1.1	1.5	0.2	2.3	0.2
Candidatus Methanoplasma	0.9	0.2	1.5	0.6	1.4	0.1	0.1	0.0	1.4	0.9	1.9	0.2	2.5	1.4	6.9	1.1	4.1	1.2

Day 7

	Ethanol		Everclear		Isopropanol		DNAzol		RNAlater		DNAgard		DESS		4C		Freezing	
Taxa ID	Average	σ	Average	σ	Average	σ	Average	σ	Average	σ	Average	σ	Average	σ	Average	σ	Average	σ
Omnitrophicaeota	35.8	1.3	48.3	6.1	41.1	2.5	0.2	0.1	7.7	1.9	0.5	0	35.4	2.1	40.4	3.0	35.2	2.2
Peptococcaceae	32.1	3.5	33.0	6.2	17.4	4.9	0	0	1.0	0.3	0.1	0.1	7.5	1.0	3.0	0.3	17.0	1.4
Smithella	3.1	0.5	1.8	0.1	6.6	0.5	0	0	0.6	0.1	0	0	6.1	0.3	4.3	0.3	4.5	0.5
Acetobacterium	3.0	0.6	3.1	0.8	3.2	0.8	0	0	0	0	0	0	3.7	0.8	3.1	0.8	2.3	0.9
Mesotoga	3.0	0.5	0.4	0.2	5.4	1.2	0	0	0.9	0.4	0.1	0.1	0.9	0.1	6.7	0.2	6.2	0.7
Methanoculleus	2.4	1.1	1.0	0.3	2.3	0.4	0	0	1.5	0.5	0.6	0.5	14.1	2.3	6.6	0.7	3.2	0.9
JS1 bacterium	2.2	0.8	0.3	0.2	2.7	2.3	0	0	0.9	0.3	0.3	0.1	5.8	1.4	3.9	0.8	6.2	1.0
Arcobacter	0	0	0	0	0	0	0	0	28.8	1.5	0	0	0	0	0	0	0	0
Shewanella	0	0	0	0	0	0	51.4	0.8	0.3	0.1	88.0	0.1	0	0	0	0	0	0
Pseudomonas	0	0	0	0	0	0	48.1	0.9	54.0	4.6	7.1	0.3	0	0	0	0	0	0

Day 14

	Ethanol		Everclear		Isopropanol		DNAzol		RNAlater		DNAgard		DESS		4C		Freezing	
Taxa ID	Average	σ	Average	σ	Average	σ	Average	σ	Average	σ	Average	σ	Average	σ	Average	σ	Average	σ
Methanoculleus	4.1	1.1	3.3	0.1	3.0	0.7	0	0	0.6	0.1	0.3	0.1	14.3	0.5	13.2	1.0	5.2	0.6
JS1-2	10.1	4.1	10.7	1.8	21.5	2.3	0.1	0	2.0	0.8	0.3	0	11.0	1.0	7.3	1.4	7.7	0.4
Arcobacter	0.1	0	0	0	0	0	0	0	58.4	1.7	0	0	0	0	0	0	0	0
Peptococcaceae	19.3	0.7	12.5	0.6	8.8	0.6	0.1	0	0.7	0.3	0.2	0.1	5.3	0.4	6.1	0.2	16.5	3.1
Omnitrophicaeota	31.3	1.3	45.5	3.2	27.2	1.8	0.5	0	4.5	0.4	0.4	0.2	38.7	0.3	34.8	1.7	33.7	1.0
Hyphomonas	0	0	0	0	0	0	0	0	0	0	0	0	0	0	0	0	0	0
Smithella	6.4	0.7	7.6	0.7	9.2	0.8	0.1	0.1	0.8	0.1	0	0	4.9	0.4	3.8	0.6	4.1	0.7
Shewanella	0	0	0.1	0	0.1	0.1	73.4	1.2	1.2	0.1	88.2	1.4	0	0	0.6	0.2	0	0
Pseudomonas	0.1	0	0	0.1	0.1	0.2	25.6	0.8	25.4	1.2	7.4	0.6	0	0	0.1	0	0	0
Mesotoga	5.4	1.6	2.2	0.5	7.5	0.4	0.1	0.1	1.3	0.2	0.1	0	1.4	0.5	6.4	0.5	3.5	0.1

Day 28

	Ethanol		Everclear		Isopropanol		DNAzol		RNAlater		DNAgard		DESS		4C		Freezing	
Taxa ID	Average	σ	Average	σ	Average	σ	Average	σ	Average	σ	Average	σ	Average	σ	Average	σ	Average	σ
Omnitrophicaeota	18.9	4.4	27.7	2.4	14.6	0.1	1.9	0.3	5.3	0.7	1.1	0.1	30.3	1.3	4.4	0.	31.0	2.0
Acetobacterium	10.6	5.3	6.9	0.2	6.4	0.2	0.9	0.5	0.6	0.5	0.3	0.1	6.1	0.5	1.1	0	2.7	0.5
Peptococcaceae	8.6	2.5	6.0	1.1	5.9	0.4	0.1	0.1	0.7	0.1	0.3	0.1	3.5	0.1	0.3	0.2	18.4	1.1
JS1-2	5.1	1.9	9.4	2.2	20.4	4.1	0.5	0.1	1.9	0.8	0.5	0.3	7.3	0.4	1.0	0.5	11.7	0.9
Smithella	3.4	0.9	4.7	0.3	5.6	0.1	0.1	0	0.6	0.1	0	0	4.4	0.8	0.4	0.2	3.4	0.3
Methanoculleus	3.3	1.3	4.2	0.7	2.9	0.4	0.2	0.1	0.8	0.3	0.4	0.2	9.2	1.4	0.9	0.2	4.0	0.9
Mesotoga	3.2	1.0	1.9	0.4	5.1	0.8	0.2	0	2.3	0.2	0.1	0.1	1.5	0.3	0.6	0.1	2.3	0.5
Pseudomonas	0.1	0	0	0	0	0	56.3	0.3	29.8	0.3	12.1	0.5	0	0	0.4	0.2	0	0
Shewanella	0	0	0	0	0	0	30.1	1.1	1.9	0.1	62.8	2.7	0.1	0.1	86.8	0.6	0	0
Arcobacter	0	0	0	0	0	0	0	0	46.1	2.3	0	0	0	0	0	0	0	0
Hyphomonas	0	0	0	0	0	0	0	0	1.4	0.2	0	0	0	0	0	0	0	0

Table S-4. PERMANOVA statistical evaluation based on unweighted Unifrac metric according to preservative treatment.

Preservative 1	Preservative 2	Sample Size	Permutations	pseudo-F	p-value	q-value
4°C	DESS	31	999	1.343677	0.124	0.1488
4°C	DNAgard	31	999	8.301912	0.001	0.002
4°C	DNAzol	30	999	9.07923	0.001	0.002
4°C	ETOH	31	999	3.370987	0.001	0.002
4°C	Everclear	30	999	1.789876	0.025	0.034615
4°C	Freezing	30	999	1.669429	0.059	0.075724
4°C	Isopropanol	31	999	1.722807	0.042	0.056
4°C	RNAlater	30	999	5.935095	0.001	0.002
DESS	DNAgard	32	999	6.923735	0.001	0.002
DESS	DNAzol	31	999	7.698098	0.001	0.002
DESS	ETOH	32	999	3.01847	0.001	0.002
DESS	Everclear	31	999	1.295489	0.171	0.198581
DESS	Freezing	31	999	0.875648	0.55	0.576
DESS	Isopropanol	32	999	1.239426	0.183	0.205875
DESS	RNAlater	31	999	4.419482	0.001	0.002
DNAgard	DNAzol	31	999	2.130755	0.061	0.075724
DNAgard	ETOH	32	999	7.491912	0.001	0.002
DNAgard	Everclear	31	999	6.128035	0.002	0.003429
DNAgard	Freezing	31	999	5.101406	0.002	0.003429
DNAgard	Isopropanol	32	999	6.603258	0.001	0.002
DNAgard	RNAlater	31	999	5.428477	0.001	0.002
DNAzol	ETOH	31	999	7.893482	0.001	0.002
DNAzol	Everclear	30	999	6.742344	0.001	0.002
DNAzol	Freezing	30	999	5.662486	0.001	0.002
DNAzol	Isopropanol	31	999	6.885217	0.001	0.002
DNAzol	RNAlater	30	999	5.721858	0.001	0.002
ETOH	Everclear	31	999	2.450214	0.003	0.004696
ETOH	Freezing	31	999	2.604863	0.003	0.004696
ETOH	Isopropanol	32	999	2.091815	0.021	0.03024
ETOH	RNAlater	31	999	4.122246	0.001	0.002
Everclear	Freezing	30	999	0.864657	0.58	0.58
Everclear	Isopropanol	31	999	0.875452	0.56	0.576
Everclear	RNAlater	30	999	3.917349	0.002	0.003429
Freezing	Isopropanol	31	999	1.021462	0.385	0.42
Freezing	RNAlater	30	999	3.144169	0.004	0.006
Isopropanol	RNAlater	31	999	3.591872	0.001	0.002

Table S-5. Indophenol assay mirroring ammonium concentrations at Day 7.

Preservative	OD_{635}, UPC	OD_{635}, Treated PW
Ethanol	0.30 ± 0.002	0.36 ± 0.003
Everclear	0.29 ± 0.008	0.25 ± 0.005
Isopropanol	0.29 ± 0.001	0.29 ± 0.003
DNAzol	0.29 ± 0.002	0.09 ± 0.002
RNAlater	0.32 ± 0.007	2.91 ± 0.03
DNAgard	0.30 ± 0.01	0.13 ± 0.004
DESS	0.32 ± 0.003	0.07 ± 0.001
4C	0.32 ± 0.004	1.34 ± 0.02
Freezing	0.29 ± 0.03	0.37 ± 0.01

Figure S-1. NMDS (A, B) and taxonomic heat maps (C, D) of samples treated with non-proprietary preservatives: isopropanol (A, C; stress value = 9.4 x 10^{-5}) and Everclear (B, D; stress value = 9.7 x 10^{-5}). Organism identities were assigned to the highest taxonomic assignment identified. Color intensity increases with an increased relative abundance of specific taxa.

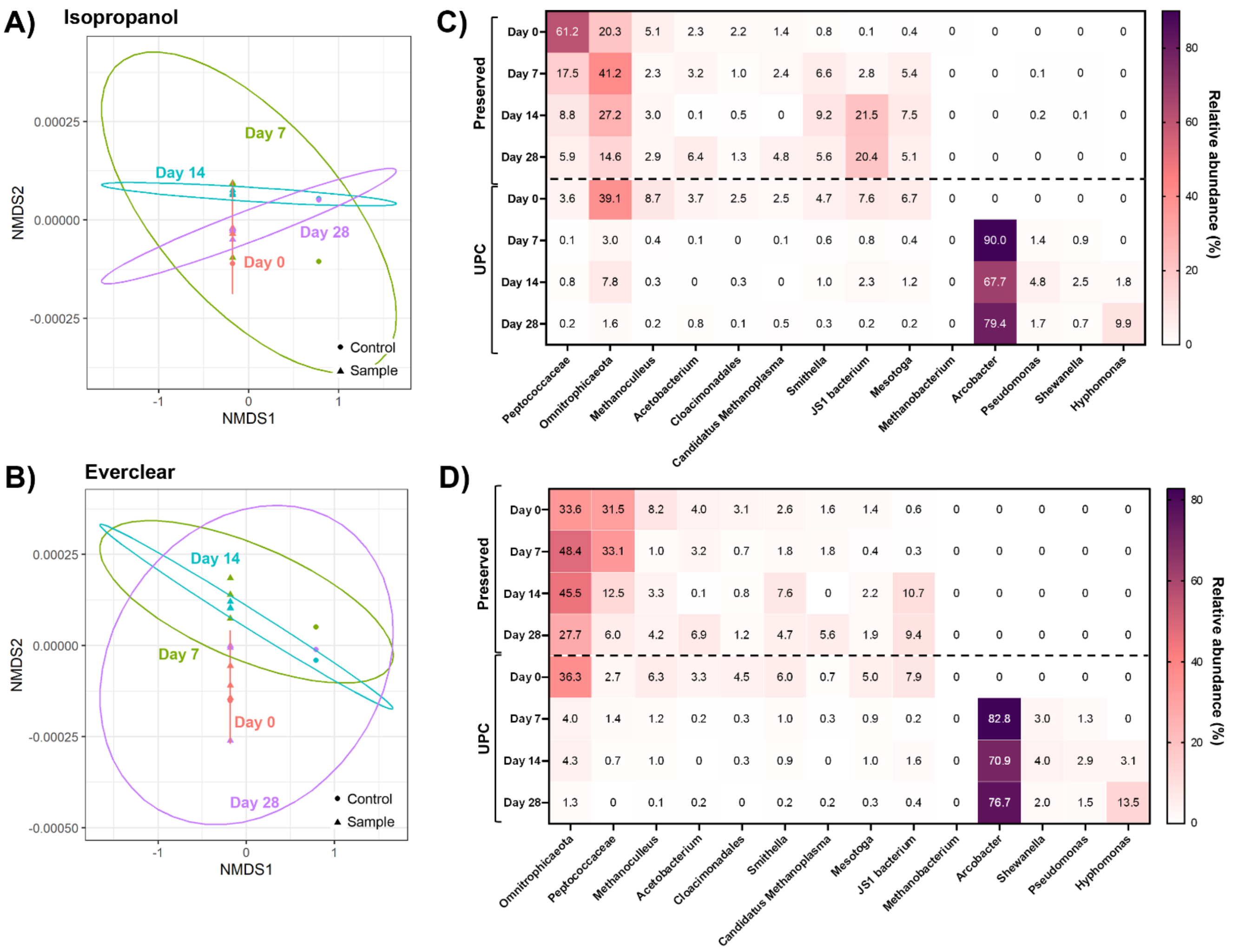

Figure S-2. NMDS (A, B; stress value = 9.1 x 10^{-5}) and taxonomic heat maps (C) of samples stored at 4°C. A) Day 7, 14, and 28 time points of UPCs and preserved samples samples are tightly superimposed, and are indentified collectively as “UPCs” and “samples” in the NMDS, respectively. B) NMDS locations have been zoomed to show tight clustering of UPC and samples. Organism identities were assigned to the highest taxonomic assignment identified. Color intensity increases with an increased relative abundance of specific taxa.

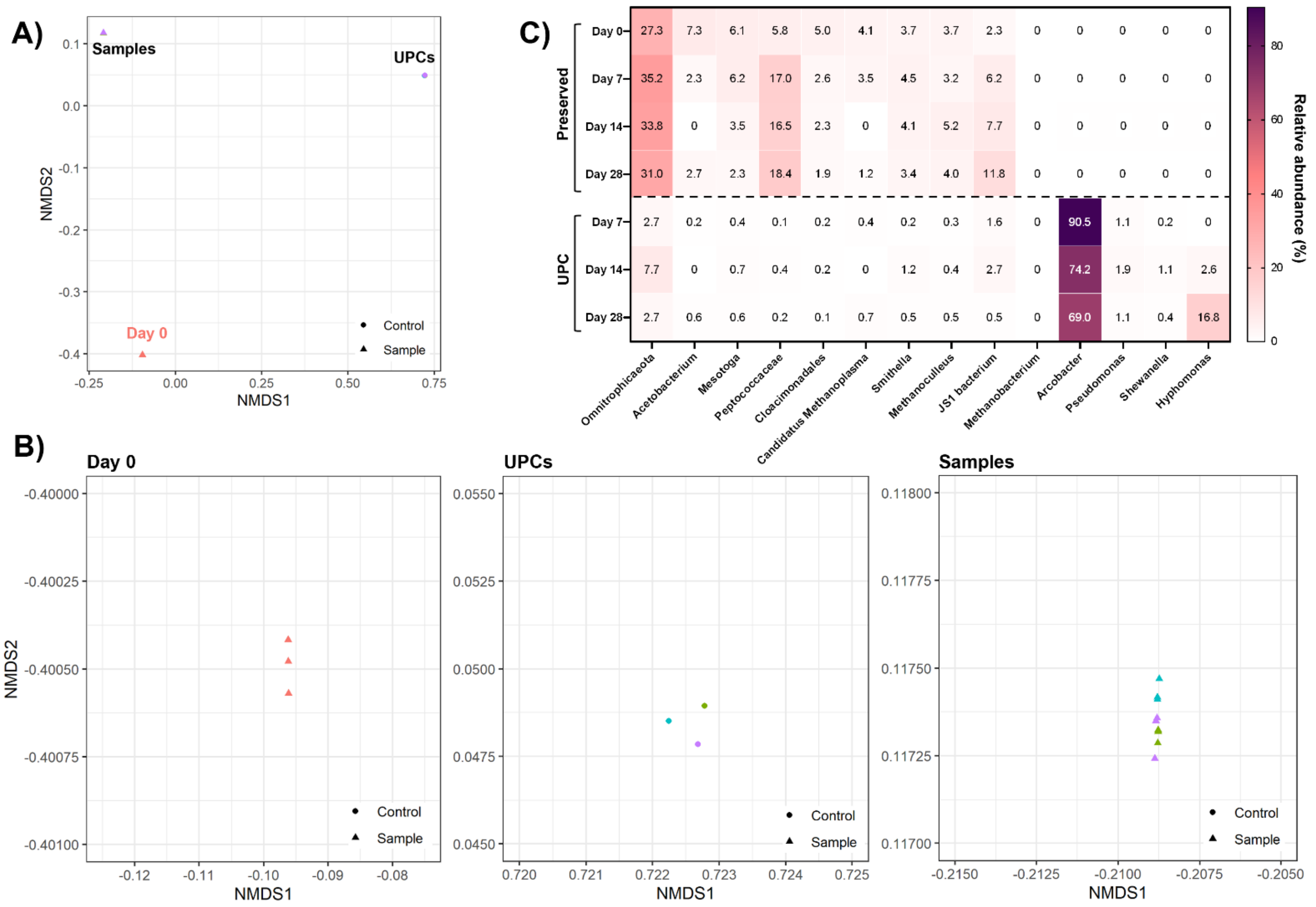

Figure S-3. NMDS (A, B; stress value = 9.9 x 10^{-5}) and taxonomic heat maps (C) of samples stored at -20°C. A) Day 0, 7, and 14 time points of UPCs and preserved samples samples are tightly superimposed, and are indentified collectively as “UPCs” and “samples” in the NMDS, respectively. B) NMDS locations have been zoomed to show tight clustering of UPC and samples. Organism identities were assigned to the highest taxonomic assignment identified. Color intensity increases with an increased relative abundance of specific taxa.

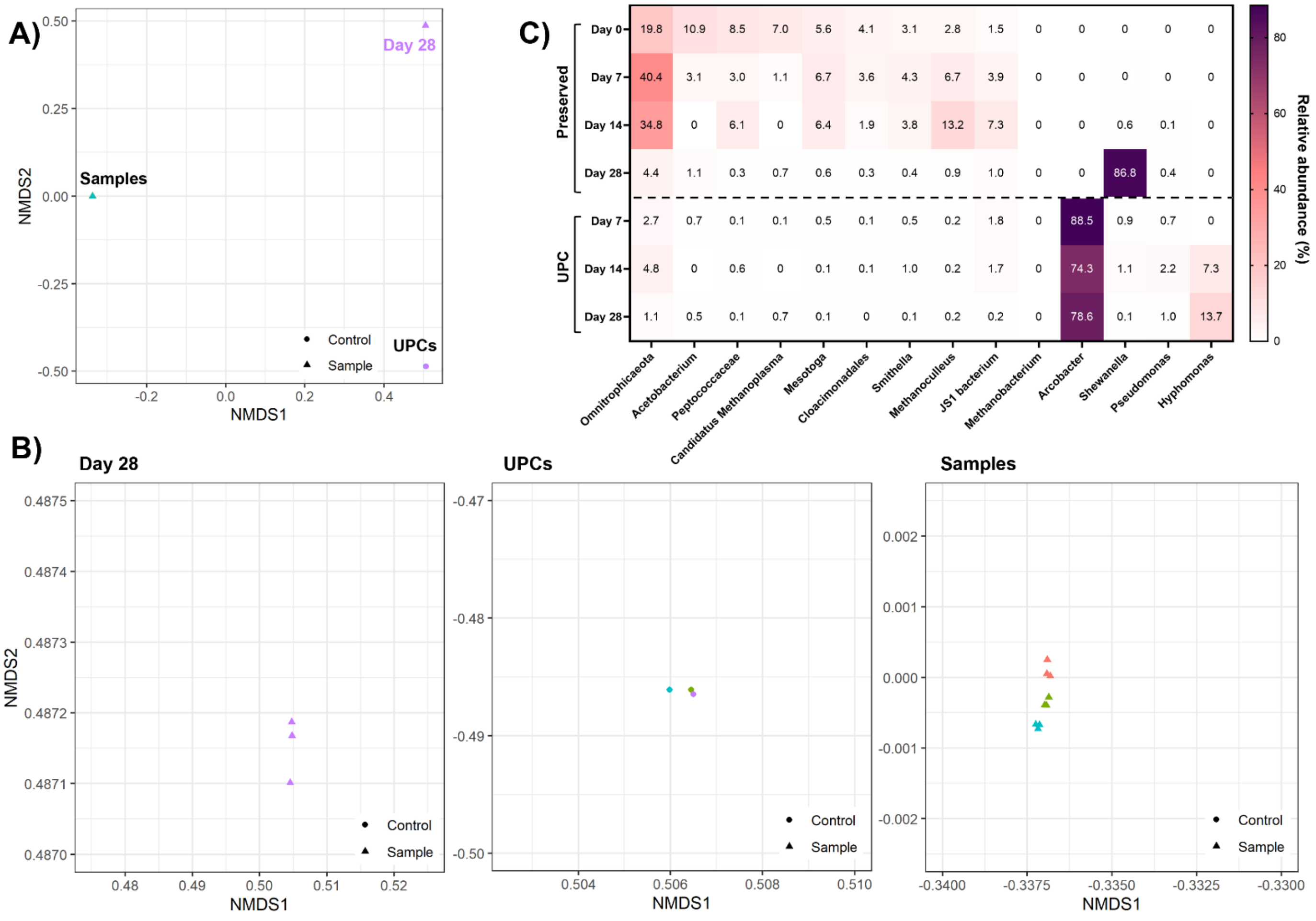

Figure S-4. NMDS (A, B; stress values = 8.8×10^{-5}, 1.0×10^{-3}, respectively) and taxonomic heat maps (C, D) of samples treated with DNAzol and DNAgard. Organism identities were assigned to the highest taxonomic assignment identified. Color intensity increases with an increased relative abundance of specific taxa.

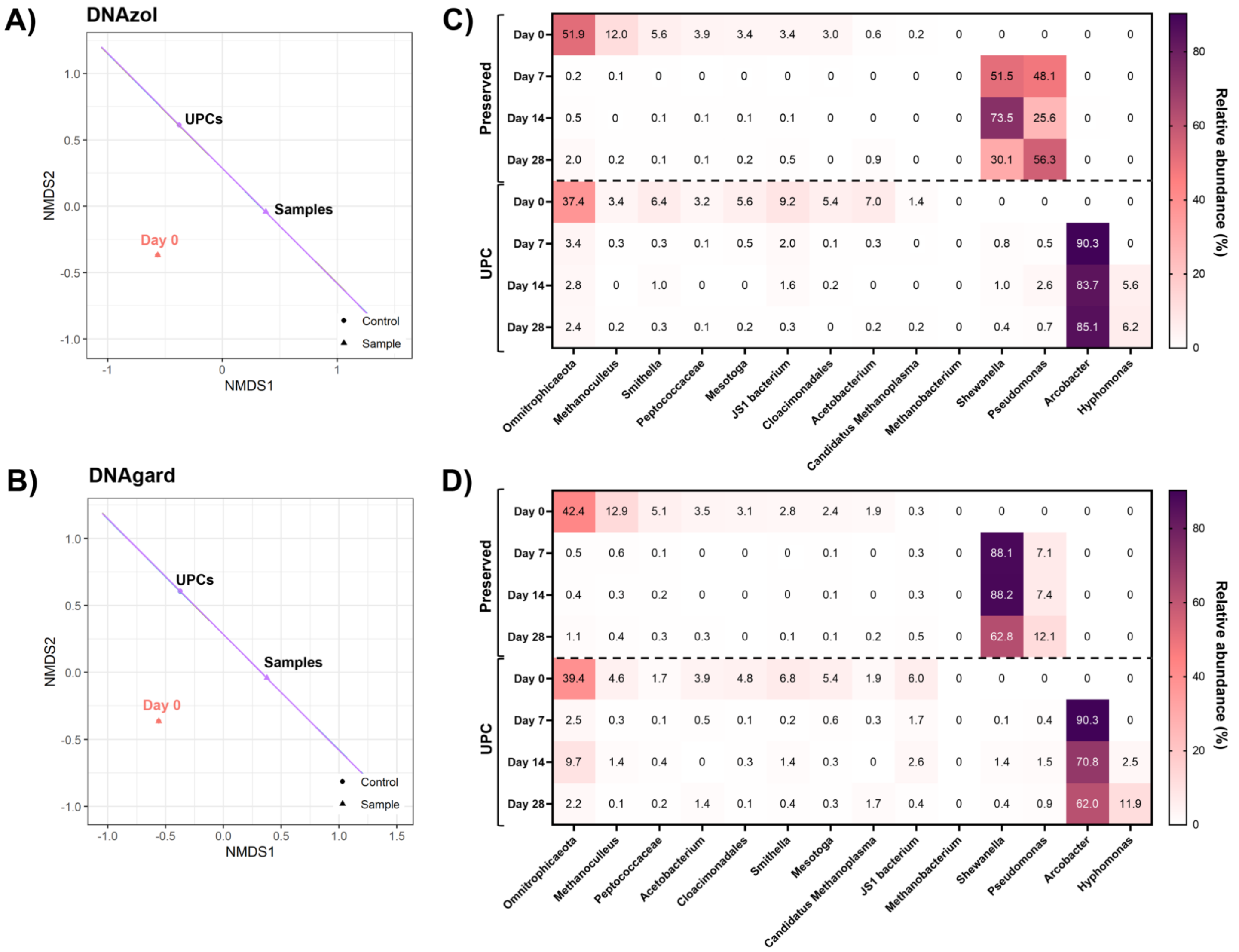

Supplement: Supplementary file 1 [file Data_Sheet_1.pdf]
